# One-Pot Synthesis of W_2_C/WS_2_ Hybrid Nanostructures for Improved Hydrogen Evolution Reactions and Supercapacitors

**DOI:** 10.3390/nano10081597

**Published:** 2020-08-14

**Authors:** Sajjad Hussain, Iqra Rabani, Dhanasekaran Vikraman, Asad Feroze, Muhammad Ali, Young-Soo Seo, Hyun-Seok Kim, Seung-Hyun Chun, Jongwan Jung

**Affiliations:** 1 Hybrid Materials Center (HMC), Sejong University, Seoul 05006, Korea; shussainawan@gmail.com; 2 Department of Nano and Advanced Materials Engineering, Sejong University, Seoul 05006, Korea; iqra.rabani@yahoo.com (I.R.); ysseo@sejong.ac.kr (Y.-S.S.); 3 Division of Electronics and Electrical Engineering, Dongguk University-Seoul, Seoul 04620, Korea; v.j.dhanasekaran@gmail.com (D.V.); hyunseokk@dongguk.edu (H.-S.K.); 4 Department of Physics, Sejong University, Seoul 05006, Korea; asadferoze55@gmail.com (A.F.); schun@sejong.ac.kr (S.-H.C.); 5 Center of Research Excellence in Nanotechnology (CENT), King Fahd University of Petroleum and Minerals (KFUPM), Dhahran 31261, Saudi Arabia; muhammadaliaskari@gmail.com

**Keywords:** hybrid, HER, WS_2_, W_2_C, symmetric, supercapacitors

## Abstract

Tungsten sulfide (WS_2_) and tungsten carbide (W_2_C) are materialized as the auspicious candidates for various electrochemical applications, owing to their plentiful active edge sites and better conductivity. In this work, the integration of W_2_C and WS_2_ was performed by using a simple chemical reaction to form W_2_C/WS_2_ hybrid as a proficient electrode for hydrogen evolution and supercapacitors. For the first time, a W_2_C/WS_2_ hybrid was engaged as a supercapacitor electrode and explored an incredible specific capacitance of ~1018 F g^−1^ at 1 A g^−1^ with the outstanding robustness. Furthermore, the constructed symmetric supercapacitor using W_2_C/WS_2_ possessed an energy density of 45.5 Wh kg^−1^ at 0.5 kW kg^−1^ power density. For hydrogen evolution, the W_2_C/WS_2_ hybrid produced the low overpotentials of 133 and 105 mV at 10 mA cm^−2^ with the small Tafel slopes of 70 and 84 mV dec^−1^ in acidic and alkaline media, respectively, proving their outstanding interfaced electrocatalytic characteristics. The engineered W_2_C/WS_2_-based electrode offered the high-performance for electrochemical energy applications.

## 1. Introduction

To overcome the ever-increasing energy necessities, researchers have devoted considerable attention to designing and developing new and eco-friendly materials for electrochemical energy production and storage uses [1,2]. Among the various electrochemical storage devices, supercapacitors (SCs) are highly favored, owing to their quick charge–discharge ability, great power density, robust cycling constancy, and simple configuration [3]. SCs are mainly divided into pseudocapacitive and electrical double-layer capacitive (EDLC) behaviors. In the EDLC, energy is filled by the accretion of ions at the junction of electrode/electrolyte. In pseudocapacitors, storage operation is ensued by rapid revocable Faradaic operation between the electro-active species of electrolyte and electrode [3,4,5]. The commonly used electrode materials for EDLCs have some limitations because of low conductivity or low specific capacitance. The improved capacitance and energy density are to be attained from pseudocapacitive property, owing to the rapid Faradic reaction. On the other hand, one of the water electrolysis processes of hydrogen evolution reaction (HER) is a prominently accomplished route for green energy. The efficient HER electrocatalysts are improve the rate of electrolysis and produce low overpotential, to reach a specific current density [6,7]. Precious platinum (Pt) is a highly proficient HER electrocatalyst, but costliness and low stability obstruct its commercial use [8,9]. Due to the economic issue, researchers are keen on developing inexpensive and earth-abundant electrode materials [3,10,11].

Transition metal dichalcogenides (TMD) materials, especially metal sulfides (MS_2_, M = W, Mo, Co, etc.) are received greater consideration for energy storage and HER devices because of its many beneficial properties such as covalently bonded S−M−S with feeble van der Waals relations between the each layer, efficient mass transport, high specific area, robust edges, and chemical stability [1,12,13]. Moreover, the layered 2D TMD materials own excessive potential as the SC electrode materials, due to their excellent electronic structure, rapid ion intercalation, and preferred pseudocapacitive behavior. However, the low intrinsic conductivity and inactive basal planes are greatly limited to their widespread use [14,15]. In order to circumvent the electrical conductance limitations and to enhance the structural stability and efficiency, TMDs can be hybridized with other high-conducting materials. Carbonaceous materials (graphene or CNT) and transition metal carbides (TMCs) are suitable partner materials for hybridization. TMCs, such as Mo_2_C and W_2_C, which are newer than graphene and CNT, could be useful electrodes for supercapacitors and HER [16,17,18]; however, their performance was not widely appealing. The previous reported hybrid materials are MoSe_2_/Mo_2_C [9], WS_2_/reduced graphene oxide hybrids [14,19], MoS_2_/Ti_3_C_2_T_x_ hybrid [20], MoS_2_/WS_2_/graphene heterostructures [21], MoS_2_/Mo_2_C hybrid nanosheets [22], WS_(1-x)_Se_x_ decorated 3D graphene [12], graphene supported lamellar 1T′-MoTe_2_ [23], and MoS_2_/reclaimed carbon fiber [24]. Recently, the W_2_C/WS_2_ has been used as a potential candidate to perceive the enriched catalytic properties for high hydrogen evolution characteristics [25,26]. Furthermore, Wang et al. [27] fabricated W_x_C@WS_2_ heterostructure via carbonizing WS_2_ nanotubes, which produced the overpotential of 146 mV at 10 mA cm^−2^ and Tafel slope of 61 mV dec^−1^. Chen et al. [28] also claimed the Tafel slope of 59 mV dec^−1^ with overpotential of 75 mV at 10 mA cm^−2^ in alkaline medium for a novel eutectoid-structured WC/W_2_C heterostructure. The detailed literatures are suggested to combine the W_2_C and WS_2_ and thereby to progress the electronic and electrochemical properties of resulted hybrid material.

Hence, in this work, W_2_C was chosen as a partner material for the hybridization with WS_2_, to form W_2_C/WS_2_ hybrid. This study focused on a simple one-pot strategy to synthesize W_2_C/WS_2_ as efficient and durable electrodes for electrochemical HER and SCs. So far, few reports are available for W_2_C/WS_2_ electrocatalysts for HER [26,27,29]. W_2_C/WS_2_ hybrid, for the first time, is employed as an SCs electrode in this work for improved storage behavior. W_2_C/WS_2_ possessed an excellent specific capacitance of ~1018 F g^−1^ at 1 A g^−1^ and high cycling stability with 94% retention. Furthermore, symmetric W_2_C/WS_2_ supercapacitor owned the high energy density of 45.5 Wh kg^−1^ at a low power density of 0.5 kW kg^−1^. As HER electrocatalyst, low overpotential of 133 and 105 mV was exhibited in acidic and alkaline media, respectively. Synergetic chemical coupling effects between the conducting W_2_C and semiconducting WS_2_ are believed to contribute significantly improving the electrochemical properties.

## 2. Materials and Methods 

### 2.1. Synthesis of WS_2_ and W_2_C Nanostructures

For WS_2_ synthesis, 0.5 g tungsten chloride (WO_3_) was dispersed in the 2:1 volume ratio of ethanol and DI water mixture, and then the solution was stirred for 30 min, using a magnetic stirrer. Then, 1 g of thiourea was dissolved in aqueous solution, blended, and placed on a hot plate, at 90 °C, with vigorous stirring for 2 h, and then endorsed, to realize the room temperature. Consequently, the dark solution was segregated out by centrifuge, and sediment was cleansed with deionized (DI) water and ethanol and parched at 100 °C, in oven, overnight. Lastly, the synthesized powder was sulfurized, using CVD tubular furnace using argon (Ar) carrier gas at 600 °C for 120 min.

The reported simple chemical reduction route was employed to produce the W_2_C nanoparticles [18]. Briefly, 1 g of commercial W_2_C powder was dispersed in the ethanol solution (50 mL), with stirring for 3 h, at room temperature. Subsequently, 25 mL of ammonia liquid was poured in the bath mixture and kept to vigorous stirring at 85 °C for 5 h. Then, resulted sediment, after being cleansed using DI water by centrifuge, was kept in an oven, overnight, at 60 °C. The product powder was kept under the gas mixture of H_2_ (80 standard cubic centimeters per minute, sccm), CH_4_ 50 (sccm), and Ar environment in the tubular furnace at 850 °C for 3 h annealing process. Finally, the W_2_C powder was collected after the tube attained room temperature.

### 2.2. Synthesis of W_2_C/WS_2_ Hybrids

**Figure 1:**
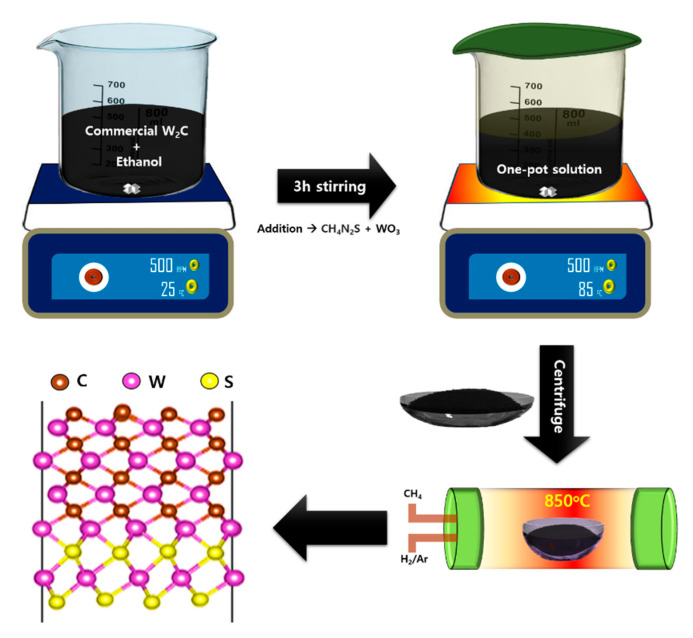
Graphic representation for the synthesis of W_2_C/WS_2_ hybrid with its derived structure.

About 1 g of commercial W_2_C powder (Sigma Aldrich, Seoul, Korea; CAS number: 12070-12-1) was dispersed in the ethanol solution (50 mL), with stirring, at room temperature, for 3 h. Then 0.25 g WO_3_ (Sigma Aldrich, Seoul, Korea; CAS number: 1314-35-8) was dispersed in ethanol and DI water mixture solution. Then 0.5 g of thiourea (Sigma Aldrich, Seoul, Korea; CAS number: 62-56-6) dissolved aqueous solution was blended with W_2_C solution and stirred. After that, 2 mL of hydrazine solution and 30 mL of liquid ammonia were mixed with the one-pot solution and stirred for 5 h at 85 °C. The deposit was parted, cleansed with DI water, and dehydrated in a hot oven, overnight, at 60 °C. The powder was post-annealed in a furnace, at 850 °C, for 3 h, under the CH_4_ (50 sccm), H_2_ (80 sccm), and Ar atmosphere. Figure 1 shows the illustration for the synthesis of a hierarchically W_2_C/WS_2_ hybrid nanostructure.

### 2.3. HER Performance

For the working electrode preparation, polyvinylidene fluoride (PVDF), active material (W_2_C, WS_2_, and W_2_C/WS_2_), and carbon black at 10:80:10 mass ratio were mixed, and *N*-methyl-2-pyrrolidone (NMP) was added drop-wise. The paste was layered on Ni foam (NF) and overnight dehydrated at 100 °C. For the reference electrode, Ag/AgCl and Hg/HgO were used in acidic and alkaline media performance, respectively, with a graphite counter electrode. We recorded iR corrected linear sweep voltammetry (LSV) by using an electrochemical system (model: 660D; company: CH Instruments, Inc., Austin, TX, USA) in 1 M KOH and 0.5 M H_2_SO_4_ media, with a scan speed of 10 mV s^−1^. Electrochemical impedance spectroscopy (EIS) results were noted at the frequencies of 0.01 Hz–100 kHz in acid and alkaline media. The HER potential values were converted for reversible hydrogen electrode (RHE) by the following formula: E (vs. RHE) = E (vs. Ag/AgCl) + E^0^ (Ag/AgCl) + 0.0592 × pH for acidic medium and E (vs. RHE) = E (vs. Hg/HgO) + E^0^ (Hg/HgO) + 0.0592 × pH for alkaline medium.

### 2.4. Supercapacitor Performance

A biologic science instrument (SP150, Seyssinet-Pariset, France) was used to analyze the supercapacitor properties. The fabrication process for working electrode was same as for HER. The electrochemical performance was employed in 2 M KOH aqueous for three-electrode (denoted as half-cell) and two-electrode (denoted as symmetric) measurements. The active materials (W_2_C, WS_2_, and W_2_C/WS_2_) loaded NFs were employed as the working electrodes, along with Ag/AgCl as a reference electrode and a Pt wire as a counter electrode for three electrode measurements. For symmetric, a cut of Whatman filter paper was socked for 2 h in 2 M KOH and then dried, to remove the excess water. As-prepared filter paper was positioned between the couple of similar W_2_C/WS_2_ electrodes and pressed to make a sandwich structure. Cyclic Voltammetry (CV) and galvanostatic charge–discharge (GCD) scans were noted from −0.8 to + 0.2 V (vs. Ag/AgCl), at different sweep rates and current densities. The capacitance (*C*, F g^−1^), energy density (*E*, Wh kg^−1^), and power density (*P*, Wkg^−1^) were derived by using the Equations (1)–(3), respectively [3,30].

(1) C=(I×Δt)(m×ΔV)

(2) E=(C×ΔV2)(2×3.6)

(3) P=(E×3600)/Δt

where *m* is the mass, *I* is the current, Δ*t* is the discharging time, and Δ*V* is the potential. EIS studies were accomplished at 10 mV AC amplitude, in an open circuit, in the frequency region of 0.01 Hz to 200 KHz.

### 2.5. Characterization Details

Field-emission scanning electron microscopy (FESEM) (HITACHI S-4700, Tokyo, Japan) was used to explore the morphological studies. The atomic structures were analyzed by a JEOL-2010F transmission electron microscopy (TEM) with an operation voltage of 200 keV. The Raman spectroscopy (Renishaw inVia RE04, Gloucestershire, UK) measurements were performed in ambient conditions, using the 512 nm Ar laser source with a laser spot size of 1 µm and a scan speed of 30 s. The structural properties were characterized by Rigaku X-ray diffractometer (XRD) (Tokyo, Japan) with Cu-K_α_ radiation (0.154 nm), at 40 kV and 40 mA, in the scanning range of 10–80° (2θ). For chemical composition and binding energy, the X-ray photoelectron spectroscopy (XPS) measurements were carried out by using an Ulvac PHI X-tool spectrometer (Kanagawa, Japan) with Al K_α_ X-ray radiation (1486.6 eV). A Brunauer–Emmet–Teller (BET) study to calibrate the surface area of nanostructures was performed, using a N_2_ adsorption/desorption medium at 77 K (Micromeritics, Norcross, GA, USA). Pore size distribution was measured with the Barrett–Joyner–Halenda (BJH) analysis.

## 3. Results and Discussion

### 3.1. Materials Characteristics

**Figure 2:**
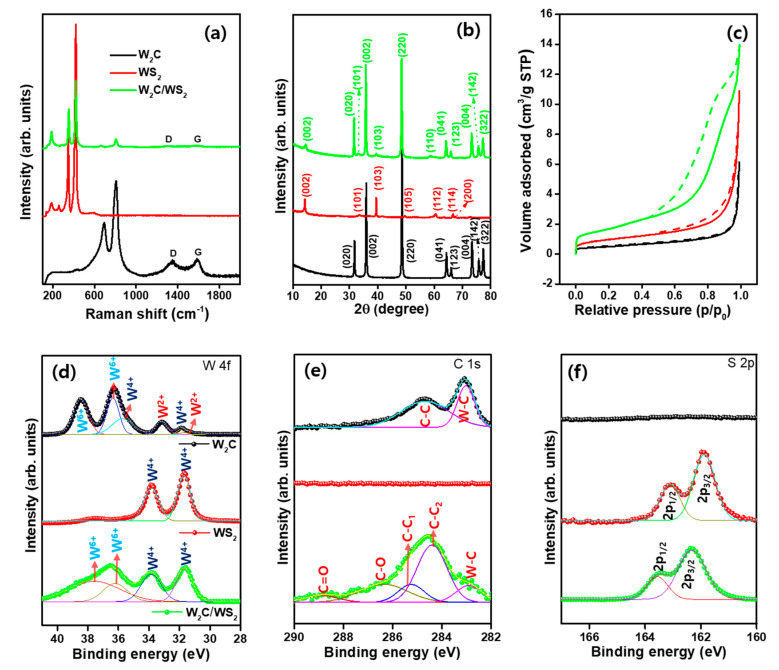
Structural studies of W_2_C, WS_2_, and W_2_C/WS_2_. (**a**) Raman profiles, (**b**) X-ray diffraction patterns, (**c**) N_2_ sorption isotherms, (**d–f**) XPS curves, (**d**) W 4f, (**e**) C 1s, and (**f**) S 2p regions.

The Raman spectroscopy were performed to measure the crystalline quality and phonon vibration mode properties of W_2_C, WS_2_, and W_2_C/WS_2_. Figure 2a displays Raman profiles for W_2_C, WS_2_, and W_2_C/WS_2_. For W_2_C, Raman spectrum reveals the solid peaks at 693 and 808 cm^−1^, which relates to the W–C mode of vibration [31,32]. The sp^2^-hybridized graphitic G and defective carbon D related bands observed at 1582 and 1353 cm^−1^, respectively [32]. For WS_2_, the characteristic bands exhibit at 354 and 420 cm^−1^, which relates to the E_2g_ and A_1g_ mode of vibration, respectively. The additional peaks in the lower frequency regions enabled at 137, 188, and 258 cm^−1^, referring to J_1_, J_2_, and A_g_ mode, support the formation of 1T′ phase WS_2_ [33,34]. Interestingly, the synchronized W_2_C and WS_2_ characteristic peaks appear for W_2_C/WS_2_ hybrid. The relatively higher intensity for A_1g_ mode than the E_2g_ mode credits to the edge exposed TMD structure, which facilitates the high electrocatalytic activity [35,36].

The materials structure was validated further by XRD. In Figure 2b, W_2_C spectrum shows polycrystalline structure. The (020), (002), (220), (041), (123), (004), (142), and (322) lattice directions observe at 31.5°, 35.1°, 48.8°, 64.1°, 65.8°, 73.2°, 75.6°, and 77.1°, respectively (JCPDS: 89-2371). The diffraction signals at 2θ values of 14.2°, 33.2°, 43.0°, 49.6°, 60.4°, 66.7°, and 67.6° correspond to the (002), (101), (103), (105), (112), (114), and (200) lattices of hexagonal WS_2_, respectively (JCPDS: 87-2417). Similar to Raman observation, XRD pattern of W_2_C/WS_2_ hybrid also produced the cumulative XRD peak positions from W_2_C and WS_2_ phases, which are indexed with black and red color, respectively. From the structural outcomes, the blended nature of W_2_C and WS_2_ phase is evidently proved in hybrid with preferential orientation of (220) and (002) lattice planes of W_2_C, which might be originated by the dominating behavior of W_2_C and their rich presence. Further, the full width at half maximum (FWHM) values were derived from the XRD peaks. The crystallite size was estimated by using FWHM by Scherrer relation [37,38]. The derived FWHM and crystallite values are provided in the Supplementary Materials Tables S1–S3 for W_2_C, WS_2_, and W_2_C/WS_2_, respectively. The estimated mean size (τ) are at 22.9, 18.7, and 18.1 nm for W_2_C, WS_2_, and W_2_C/WS_2_, respectively. The reduced-nanosize crystallites for hybrid can be originated by interconnection mechanism between W_2_C and WS_2_ and their modified crystallographic structure. The d-spacing values were estimated by the Bragg’s law (2d sinθ = nλ), and their values are well correlated with the standard results [25]. The extracted values are provided in the Supplementary Materials Tables S1–S3 for W_2_C, WS_2_, and W_2_C/WS_2_, respectively. The observed d-spacing values are considerably strained for hybrid compared with pristine, which might be due to the interfacial bonding nature.

The BET surface area was examined to assess the area and pore size distributions by using an N_2_ adsorption/desorption medium at 77 K. Figure 2c shows the N_2_ isotherms. W_2_C/WS_2_ possessed a maximum surface area of 6.368 m^2^g^−1^, equated with WS_2_ (3.405 m^2^g^−1^) and W_2_C (1.75 m^2^g^−1^). The measured total pore volume was 0.009, 0.016, and 0.020 cm^3^g^−1^ for W_2_C, WS_2_, and W_2_C/WS_2_, respectively (Supplementary Materials Figure S1). The high porosity of W_2_C/WS_2_, compared with pure, is believed to give the significant contribution of enhancing the electrocatalytic activity by promoting the electrolyte diffusion into the electrode.

XPS was employed to describe the composition and valence states of constructed material. The survey profiles are given in the Supplementary Materials Figure S2, to prove the coexistence of all the elements. The high-resolution XPS profiles for W 4f, C 1s, and S 2p states are provided in Figure 2d–f. From a W 4f XPS profile of W_2_C (Figure 2d), the deconvoluted peaks reveal the W^2+^ (31.4 and 33.1 eV), W^4+^ (31.8 and 35.69 eV), and W^6+^ (37.0 and 38.3 eV) doublets. For WS_2_, the W4f core level peaks are located at 31.7 and 33.9, due to W4f_7/2_ and W4f_5/2_, respectively. W 4f region of W_2_C/WS_2_ reveals the peaks at 31.7 eV (W4f_7/2_) and 33.8 eV (W4f_5/2_) with the W^6+^ couplets (37.6 and 36.2 eV) [39,40]. Figure 2e shows the C 1s profile and explores the graphitic sp^2^ carbon peak at 284.7 eV and W-C peak at 283.0 eV for W_2_C [6]. For the W_2_C/WS_2_ hybrid, C 1s spectrum produced the C-C_1_ (284.4 eV), C-C_2_ (285.2 eV), W-C (282.9 eV), C-O (286.3 eV), and C=O (288.9 eV) peaks [29,41,42]. Figure 2f deconvolution peaks reveal the S 2p couplets of S2p_1/2_ and S2p_3/2_ at 163.1 and 161.8 eV for WS_2_, whereas, they are at 163.6 and 162.3 eV for the W_2_C/WS_2_ hybrid_,_ respectively [43,44]. The atomic percentage of W_2_C/WS_2_ hybrid is determined to be 32.13%, 22.20%, and 45.67% for W, C, and S atoms, respectively, which is well correlated with EDX results (discussed later). The observed elemental confirmation proved the formation of W_2_C/WS_2_ hybrid.

**Figure 3:**
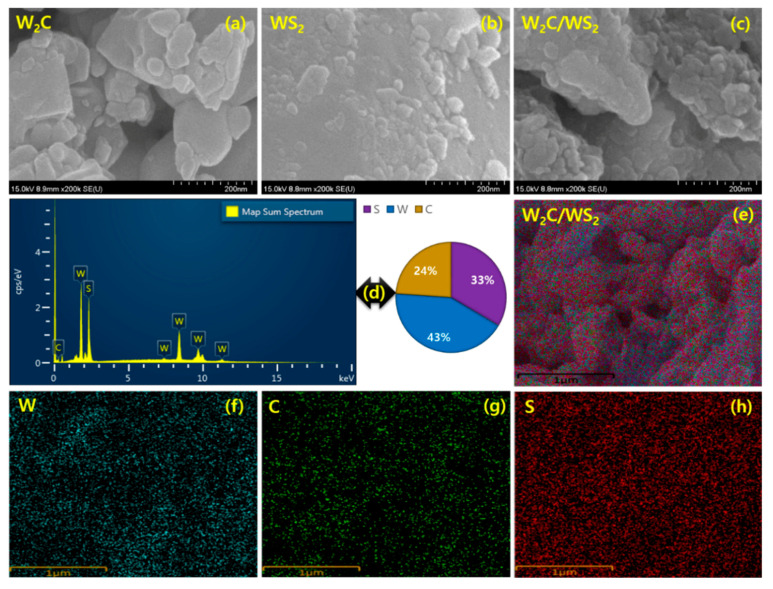
FESEM images of (**a**) W_2_C, (**b**) WS_2_, and (**c**) W_2_C/WS_2_ hybrid. (**d**) EDX spectrum of W_2_C/WS_2_ hybrid; (**e**) mapping image of W_2_C/WS_2_ hybrid and its elements, (**f**) W, (**g**) C, and (**h**) S.

Surface characteristics were further elaborated by FESEM and TEM examinations. Figure 3 shows the FESEM micrographs of W_2_C, WS_2_, and W_2_C/WS_2_. FESEM micrographs clearly picture the formation of different sizes of nanograins by chemical reduction process in the W_2_C nanoparticles (Figure 3a). Nano-spherical-shaped agglomerated grains exhibit for WS_2_ (Figure 3b). The sizes of the grains are considerably varied in the nanoscales, due to the bulk agglomeration process during the annealing. In the hybrid, the spherically shaped W_2_C particles seem to cover the WS_2_ particles (Figure 3c) due to the interconnected mechanism. Reduced sizes of the grains appear with cauliflower like agglomerated grain bunches and well-interconnected domain structure for W_2_C/WS_2_ hybrid. To prove the hybrid formation, EDX spectrum for W_2_C/WS_2_ clarifies the elemental composition, as shown in Figure 3d. Furthermore, the mapping images are provided to confirm the equal distribution of all the elements in the hybrid (Figure 3e–h).

**Figure 4:**
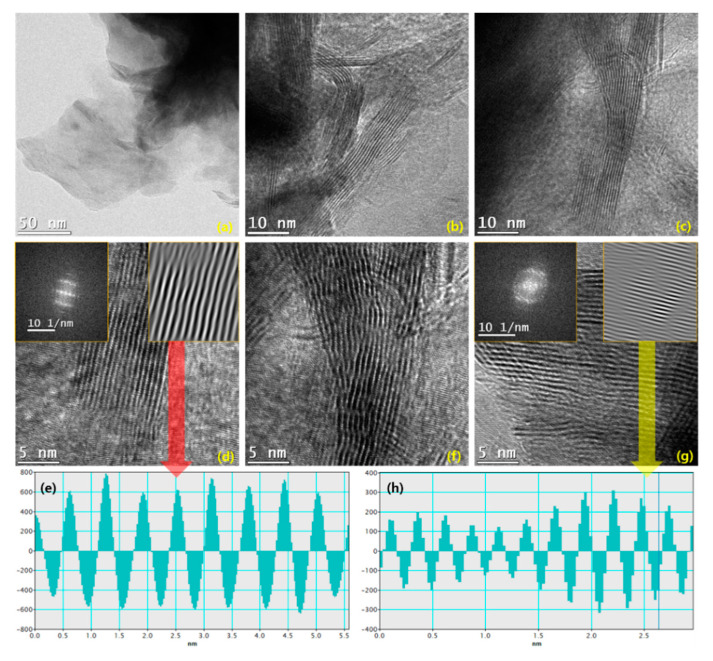
HRTEM images for W_2_C/WS_2_ hybrid. (**a**) Low- and (**b,c**) high-resolution TEM images. (**d**) Layered WS_2_ structure with the inset of FFT and iFFT patterns. (**e**) Phase profile spectrum for (002) lattice orientation of WS_2_ with 6.2 nm spacing in the W_2_C/WS_2_ hybrid. (**f,g**) High-resolution TEM images for W_2_C related portion in the hybrid with inset of FFT and iFFT patterns. (**h**) Phase profile spectrum for (020) lattice orientation of W_2_C with 2.9 nm spacing in the W_2_C/WS_2_ hybrid.

TEM measurements were carried out for W_2_C/WS_2_ hybrid (Figure 4). The different magnification TEM images are provided in Figure 4a–c. Vertically aligned nano-stirpes-like structures are broadly exhibited for the hybrid. The interconnection between the layered fringes and finger-printed structures is clearly visualized. Due to the polycrystalline lattices for W_2_C/WS_2_ hybrid, the different widths of the lattice fringes are obviously demonstrated in the TEM images (Figure 4b,c). A higher magnification TEM image (Figure 4d) explores the layer structure with the cross-section of different lattice fringes in the W_2_C/WS_2_ hybrid (inset—fast Fourier transform (FFT), left panel). The phase profile spectrum, extracted by point mask mode and inverse FFT (iFFT, right panel) pattern of inset Figure 4d, shows 6.2 nm spacing which related to (002) WS_2_ lattice orientation (Figure 4e). The fingerprint structured grains interface with layered WS_2_ (Figure 4f–g) [44]. The phase profile spectrum, extracted from the iFFT pattern of inset Figure 4g, elevates 2.9 nm spacing, which is related to (020) W_2_C lattice orientation (Figure 4h).

### 3.2. Hydrogen Evolution Studies

**Figure 5:**
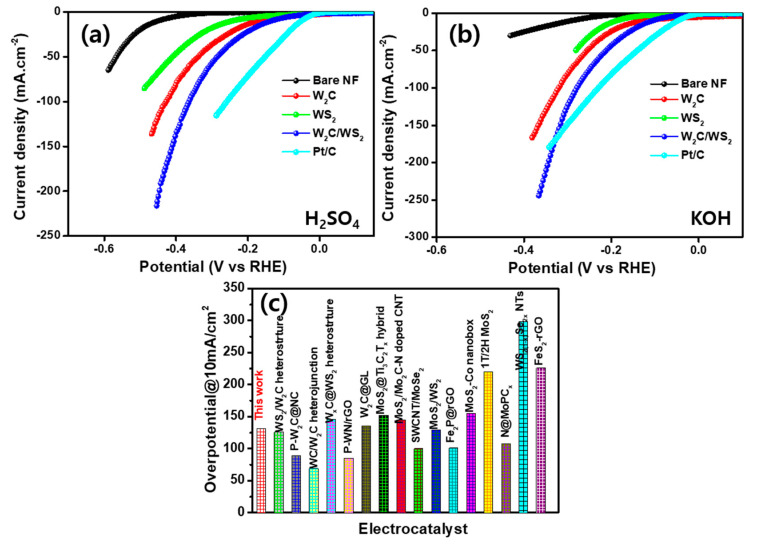
(**a**,**b**) Hydrogen evolution polarization profiles for Pt/C, bare NF, W_2_C, WS_2_, and W_2_C/WS_2_ at 10 mV s^−1^ sweep speed in (**a**) 0.5 M H_2_SO_4_ and (**b**) 1 M KOH media. (**c**) Overpotential comparison of different electrocatalysts.

Active-materials-coated NFs were engaged as working electrodes to appraise the HER activities in 0.5 M H_2_SO_4_ and 1 M KOH electrolytes, at room temperature. Figure 5a explores iR-recompensed LSV polarization profiles in 0.5 M H_2_SO_4_, using 10 mV s^−1^ sweep speed. The W_2_C and WS_2_ produce 171 and 242 mV to attain 10 mA cm^−2^, respectively. In contrast, the W_2_C/WS_2_ hybrid produces an overpotential of 133 mV at 10 mA cm^−2^ (51 mV @ 10 mA cm^−2^ for Pt/C). The exhibited low overpotential credits to interfacial active edges sharing and rapid electron conductivity in the W_2_C/WS_2_ which proves the importance of hybrid formation. In the 1 M KOH media (Figure 5b), the W_2_C/WS_2_ electrode also produces highly dynamic HER behavior with a small overpotential of 105 mV at 10 mA cm^−2^ than WS_2_ (189 mV) and W_2_C (123 mV). The HER performance of W_2_C/WS_2_ is superior to most of the hybrid-based electrodes (Figure 5c) [45,46,47,48]. Li et al. [29] have prepared the nanocomposite of N, S-decorated porous carbon matrix encapsulated WS_2_/W_2_C (WS_2_/W_2_C@NSPC), which delivered the small overpotential of 126 and 205 mV in 0.5 M H_2_SO_4_ and 1.0 M KOH, respectively. In addition, Nguyen et al. [26] have reported the 170 mV of onset potential with 55.4 mV dec^−1^ of Tafel slope in 0.5 M H_2_SO_4_ for W_2_C@WS_2_ nanoflowers synthesized by hydrothermal method.

**Figure 6:**
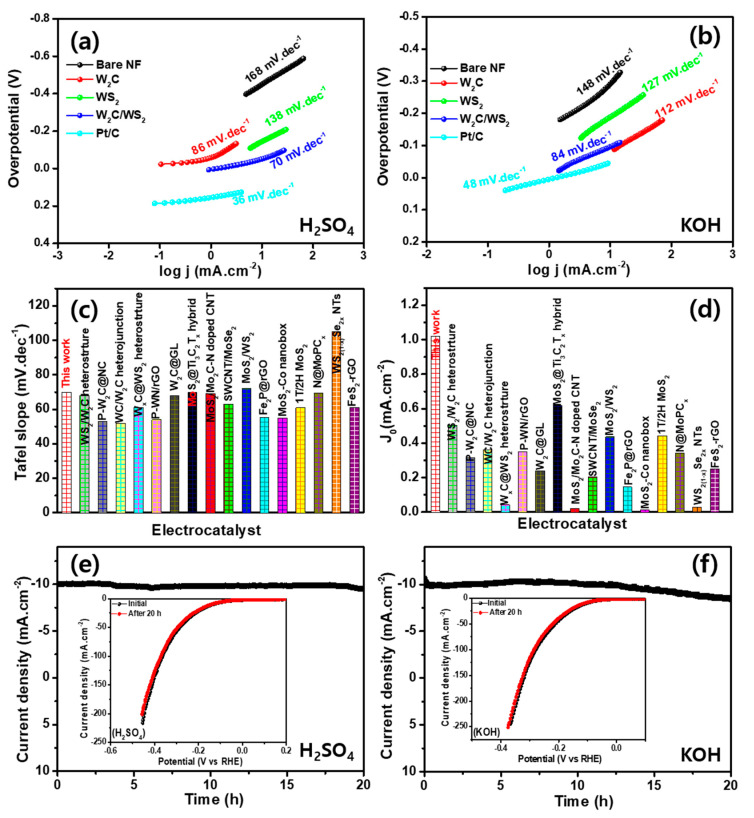
(**a**,**b**) Tafel plots for Pt/C, bare NF, W_2_C, WS_2_, and W_2_C/WS_2_ hybrid at 10 mV s^−1^ sweep speed in (**a**) 0.5 M H_2_SO_4_ and (**b**) 1 M KOH media; comparison of (**c**) Tafel slope and (**d**) exchange current density with different electrocatalysts; chronoamperometric profile of W_2_C/WS_2_ hybrid for 20 h continuous hydrogen evolution reaction (HER) operation in (**e**) 0.5 M H_2_SO_4_ and (**f**) 1 M KOH electrolyte (inset: LSV curves before and after 20 h operation).

Tafel slope is a factor to indicate the inherent electrocatalytic activity of the electrode. The Tafel slope values of Pt/C, bare NF, W_2_C, WS_2_, and W_2_C/WS_2_ electrocatalysts, are 36, 168, 86, 138, and 70 mV dec^−1^, respectively, in H_2_SO_4_ medium (Figure 6a). The outputs prove the outstanding electrocatalytic activity of the W_2_C/WS_2_ hybrid. Exchange current densities (j_0_) assessed by extrapolation Tafel lines to X-axis and their values observe at ~1.03, 1.02, 0.55, and 0.19 mA cm^−2^ for Pt/C, W_2_C/WS_2_, WS_2_, and W_2_C, respectively. W_2_C, WS_2_, and W_2_C/WS_2_ produce the Tafel slopes of 141, 127, and 84 mV dec^−1^, respectively, in KOH medium (Figure 6b). The small Tafel slope of W_2_C/WS_2_ also supports high HER behavior of hybrid electrode in the KOH electrolyte. The extrapolated j_0_ is 0.93, 0.72, and 0.38 mA cm^−2^ for W_2_C, WS_2_, and W_2_C/WS_2_, respectively, in KOH electrolyte. The exhibited Tafel slope range suggests that HER involves a Volmer–Heyrovsky mechanism for W_2_C/WS_2_ hybrid, with electrochemical desorption as the rate-regulatory direction [44,49,50,51]. Outstanding HER activity in the W_2_C/WS_2_ hybrid could be explained with electrode kinetics by accumulated electrocatalytic edge facets and a high ratio of charge transfer. The observed HER parameters are provided in Supplementary Materials Table S4 for all the measured electrodes. The j_0_ and Tafel values are superior to most reported hybrid electrocatalysts (Figure 6c,d and Supplementary Materials Table S5) [46,48,52]. The low overpotential, large j_0_ value, and small Tafel slope in the W_2_C/WS_2_ hybrid also confirm the importance of hybrid formation for efficient HER electrocatalytic activity in KOH and H_2_SO_4_ medium.

CV profiles were acquired in the non-Faradaic region, to estimate the double-layer capacitance (C_dl_) (Supplementary Materials Figure S3a–c). The C_dl_ by linear fitting (Supplementary Materials Figure S3c) are 3.81 mF cm^−2^ (in H_2_SO_4_) and 3.33 mF cm^−2^ (in KOH) for the W_2_C/WS_2_ hybrid. Electrochemical surface areas are 108 and 83 cm^2^ in H_2_SO_4_ and KOH, respectively. A stability and durability evaluation of W_2_C/WS_2_ electrode was carried out by using chronoamperometric response at a persistent 133 and 105 mV overpotential in H_2_SO_4_ and KOH (Figure 6e,f), respectively. No significant decline is observed over 20 h in H_2_SO_4_ the medium, whereas slight deterioration is exhibited for the KOH medium. Note the excellent robustness of W_2_C/WS_2_ electrode in the H_2_SO_4_, rather than KOH, medium for HER. LSV curves at initial and after 20 h of continuous HER operation are shown in the inset of Figure 6e,f, respectively.

To probe insights for electrocatalytic activity of materials, EIS was performed in the H_2_SO_4_ and KOH (Supplementary Materials Figure S4). The observed EIS plot revealed the low charge-transfer resistance (R_ct_) and swift electron transfer via the electrolyte–electrode interface for W_2_C/WS_2_. The charge-transfer resistances, R_ct_, of W_2_C/WS_2_ (~1.8–2.2 Ω) in the H_2_SO_4_ and KOH media are lower than those of W_2_C and WS_2_. Moreover, the small series of resistances (~1.5–2.5 Ω) of all the electrodes suggests that the active materials are well integrated with the porous NF. The high electrocatalytic properties and robust solidity of the W_2_C/WS_2_ hybrid support it as a potential material to substitute Pt in HER application.

### 3.3. Supercapacitor Performances

**Figure 7:**
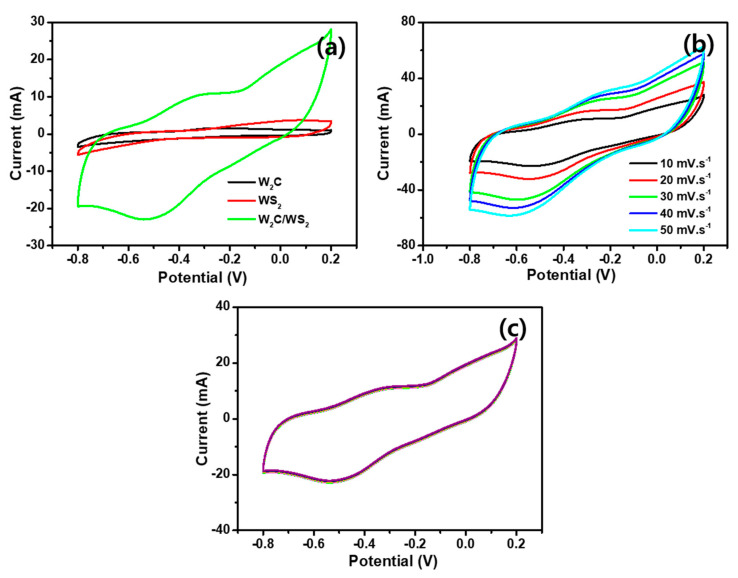
Supercapacitor Cyclic Voltammetry (CV) for three electrode measurement: (**a**) CV curves for W_2_C, WS_2_, and W_2_C/WS_2_ electrodes; (**b**) different scan rate CV curves for the W_2_C/WS_2_ hybrid; (**c**) multiple cycle CV curves for the W_2_C/WS_2_ hybrid.

Electrochemical storage properties were elucidated by CV and GCD tests in 2 M KOH electrolyte, using three electrodes, as explained in the experimental part of the manuscript. The CVs were recorded with the potential interval of −0.8 to 0.2 V vs. Ag/AgCl at 10 mV s^−1^ sweep speed for the W_2_C, WS_2_, and W_2_C/WS_2_ electrodes (Figure 7a). All the electrodes produce the identical CV loops with Faradaic-adsorptions-blended EDLC operations [53]. The W_2_C/WS_2_ hybrid electrode shows a wider electrochemical area than the W_2_C and WS_2_. Moreover, the W_2_C/WS_2_ hybrid shows a couple of redox peaks (−0.61 and −0.25 V), indicating the reversible reaction from W^4+^ to W^6+^, corresponding to the proton’s absorption/desorption into the WS_2_ interlayers [54]. Figure 7b shows CVs of various scan rates (10–50 mV s^−1^), and their shapes are maintained, indicating good electrochemical capacitive characteristics and high-rate performance. The results for WS_2_ and W_2_C at various scan speeds are shown in Supplementary Figure S5a,b, respectively. Successive 100 CV cycles were executed in the potential region of −0.8 to 0.2 V, to assess the stability for the W_2_C/WS_2_ electrode, and its output is presented Figure 7c. Due to the EDLC-combined Faradaic storage mechanism, it possesses good stability with minimal degradation over the repeated cycles [55].

**Figure 8:**
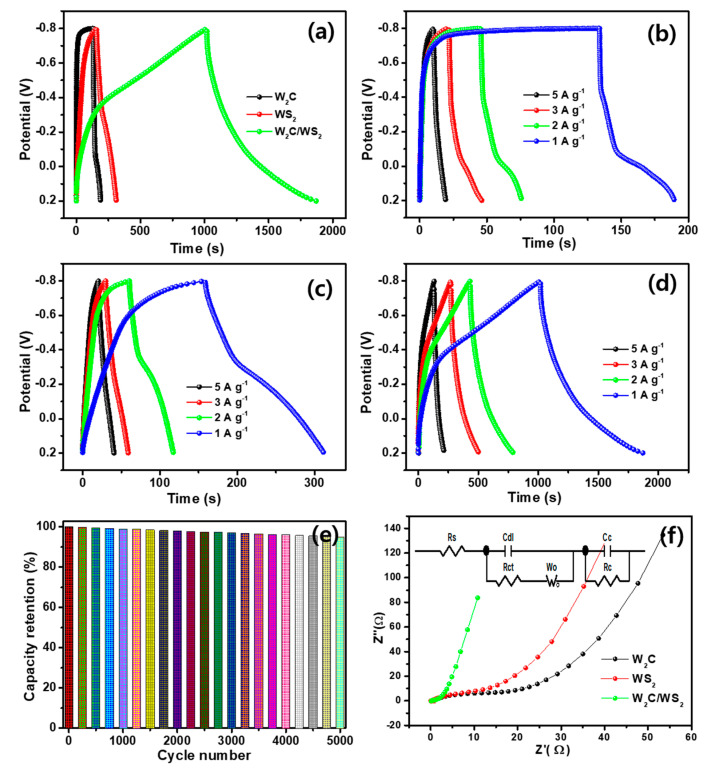
(**a–d**) Galvanostatic charge–discharge (GCD) profiles for three electrode measurement; (**a**) GCDs at 1 Ag^−1^ for W_2_C, WS_2_, and W_2_C/WS_2_; GCDs at different current densities for (**b**) W_2_C, (**c**) WS_2_, and (**d**) W_2_C/WS_2_; (**e**) stability performance of W_2_C/WS_2_ hybrid; (**f**) EIS curves for W_2_C, WS_2_, and W_2_C/WS_2_ (inset-fitted circuit).

The electrochemical storage performance was further tested by GCD curves, as shown in Figure 8a–d. The slightly distorted triangle like the GCD curve is exhibited for the W_2_C/WS_2_ and WS_2_ electrodes, due to the redox reaction. For the W_2_C electrode, a square-structured GCD curve is exhibited, with a voltage drop, due to the easy oxidation characteristic. The W_2_C/WS_2_ electrode exposes an outstanding specific capacitance (estimated by using the Equation (1)) of 1018 F g^−1^, as compare to the WS_2_ (~158 F g^−1^) and W_2_C (~133 F g^−1^), at the current density of 1 A g^−1^. GCD analysis displays similar curves at the different current densities. Observe the specific capacitances of 133, 90, 66, and 50 F g^−1^ for W_2_C (Figure 8b) and 158, 120, 87, and 80 F g^−1^ for WS_2_ (Figure 8c) at 1, 2, 3, and 5 A g^−1^ current density, respectively. For W_2_C/WS_2_, a high specific capacitance of 1018, 866, 816, and 660 F g^−1^ exhibits at the 1, 2, 3, and 5 A g^−1^ current density, respectively (Figure 8d). The specific capacitance changes with current density, as shown in Supplementary Materials Figure S6. The significant enhancement of capacitances for W_2_C/WS_2_ electrode credits to the mutual interactions between W_2_C and WS_2_, large surface area, rich active edge facets of WS_2_, and high electronic conductivity of W_2_C to enable the rapid transference of electrons during a charge–discharge process. Supplementary Materials Table S6 provides the extended comparison of W_2_C/WS_2_ SCs performance with the previously reported hybrid electrodes.

Cyclic stability is an essential property for the supercapacitor electrodes. In the case of the W_2_C/WS_2_ electrode, 94% of primary capacitance was perceived after 5000 cycles (Figure 8e), suggesting long-term stability. EIS measurements were performed to prove the charge-transfer characteristics (Figure 8f). Fitted curve from the Nyquist profile is inserted in Figure 8f, where C_dl_ is the double-layer capacitance, R_ct_ is the charge-transfer resistance, R_s_ is the series resistance, W_o_ the Warburg impedance at open circuit voltage, and R_c_ and C_c_ are the capacitive resistance and capacitive capacitance, respectively. The output curve indicates that the charge-transfer resistance significantly reduces for W_2_C/WS_2_ hybrid electrode, as compared to their pristine. A lower R_ct_ (~1.0 Ω) was obtained by the hybrid electrode, as compared to the WS_2_ (~13.9 Ω) and W_2_C (10.1 Ω), respectively.

**Figure 9:**
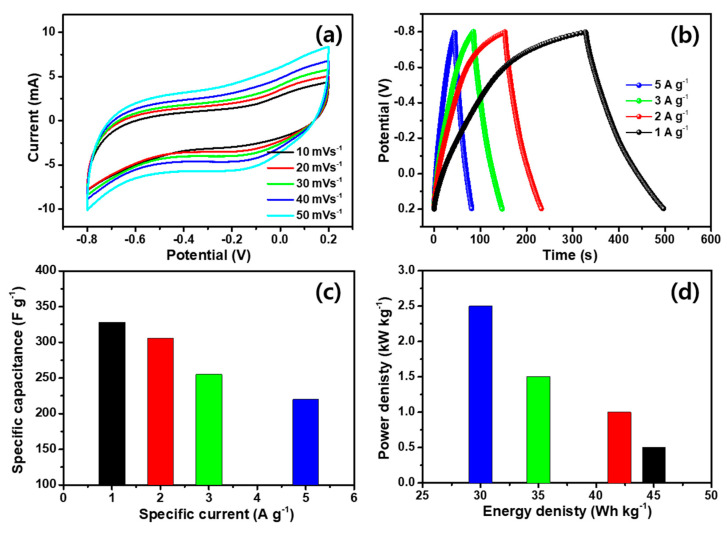
Symmetric supercapacitor performance of W_2_C/WS_2_. (**a**) Different scan rate CV curves for symmetric W_2_C/WS_2_ hybrid; (**b**) GCDs for symmetric W_2_C/WS_2_ at different current densities; (**c**) specific capacitance at various scan rates for W_2_C/WS_2_ by symmetric measurements; (**d**) Ragone plots of W_2_C/WS_2_ for symmetric device.

To assess the practical application, the symmetric supercapacitor (SSC) was assembled with two identical W_2_C/WS_2_ hybrid electrodes. The electrochemical CV measurement of SSC was measured by using the similar potential range of half-cell measurements. Figure 9a shows the CV curves for W_2_C/WS_2_ symmetric cell device. The modified rectangular shape of CV curve for the W_2_C/WS_2_ hybrid SSC reveals slightly shouldered redox reaction, confirming the key contribution of EDLC characteristics. The constructed SSC device gives an excellent current response and larger integral area, compared to previous TMDs-based materials [56,57]. The different scan rates, using performed CV curves, prove the high rate of capability of the prepared SSC devices. Figure 9b shows the different current densities, using prepared SSC GCD curves, indicating the excellent electrochemical rate capability. The charge/discharge time considerably decreases for the SSC device due to its direct intercalation and extraction of ions through the solid electrolyte, compared with its half-cell outcomes. The capacitance value of symmetric device was valued from the GCD curve, using Equation (1) [58]. The W_2_C/WS_2_ hybrid delivers the higher symmetric capacitance of 328, 306, 255, and 220 F g^−1^ at 1, 2, 3, and 5 A g^−1^ of current densities, respectively, as presented in Figure 9c. Interestingly, our symmetric device results show the enhancing capacitance, compared to other symmetric capacitor results [56,59,60]. The specific energy and specific power values are significant for the practical uses, which were weighed by relations 2 and 3, respectively. The symmetric device carries the energy densities of 45.5, 42.5, 35.4, and 30.5 Wh kg^−1^ at 0.5, 1.0, 1.5, and 2.0 kW kg^−1^ power density, respectively, as given in the Figure 9d.

The exhibited energy density of symmetric W_2_C/WS_2_ capacitor is superior to the recently reported symmetric devices using TMDs and TMCs electrodes, MoS_2_ sheets (18.43 Wh kg^−1^) [60], Ti_3_C_2_T_x_/MWCNT (3 Wh kg^−1^) [61], s-MoS_2_/CNS (7.4 Wh kg^−1^) [62], 3D-graphene/MoS_2_ (24.59 Wh kg^−1^) [63], MoS_2_/RCF (22.5 Wh kg^−1^) [24], MoS_2_/RGO/MoS_2_@Mo (6.22 Wh kg^−1^) [56], and MoS_2_ sponge (6.15 Wh kg^−1^) [59]. Overall, our findings demonstrate that the inclusion of carbide-based material with TMDs deliberately improves the conductance of the hybrid material, assists swift conveyance of electrons/ion, and improves the stability of electrode material.

## 4. Conclusions

We have successfully engineered the W_2_C/WS_2_ hybrid electrode by a simple cost-effective one-pot chemical reaction. Highly conductive W_2_C-supported WS_2_ hybrids were designed to promote high electrocatalytic activity for HER and SCs by accumulating the number of active edges and facilitating the swift electron transport. In the case of HER, the interfaced W_2_C/WS_2_ hybrid produced the small overpotentials of 133 and 105 mV, to achieve the 10 mA cm^−2^ current density with the Tafel slope of 70 and 84 mV dec^−1^ in H_2_SO_4_ and KOH media, respectively, which proved the outstanding electrocatalytic HER characteristics. Half-cell measurements unveiled the remarkable specific capacitance of ~1018 F g^−1^ at 1 A g^−1^ with the rate competency nature and robust responses for W_2_C/WS_2_ hybrid electrode. W_2_C/WS_2_-based symmetric supercapacitor exposed the specific energy of 45.5 Wh kg^−1^ at 0.5 kW kg^−1^ specific power with a capacitance of 328 F g^−1^ at 1 A g^−1^ current density. The suggested low-cost methodology of one-pot reaction is highly feasible to fabricate the efficacious nanostructured hybrids and has larger-scale production capability because of its controlled synthesis process. Hence, the developed hybrid material and methodology have a broad scope for the future electrochemical applications.

## Supplementary Materials

The following are available online at https://www.mdpi.com/2079-4991/10/8/1597/s1. Figure S1: Pore diameter versus pore volume variations for the nanostructures. Figure S2: Survey XPS spectrum of (a) W_2_C, (b) WS_2_, and (c) W_2_C/WS_2_. Figure S3: CV spectra in the non-Faradaic region with different scan rates for W_2_C/WS_2_ hybrid HER electrodes in (a) 0.5 M H_2_SO_4_ and (b) 1 M KOH electrolyte and (c) their current differences. Figure S4: EIS spectra of Pt, W_2_C, WS_2_, and W_2_C/WS_2_ hybrids electrodes in (a) 0.5 M H_2_SO_4_ and (b) 1M KOH electrolyte. Figure S5: CV curves for the (a) WS_2_ and (c) W_2_C electrodes at various scan rates, using half-cell (10–50 mVs^−1^). Figure S6: The specific capacitance variations at different current densities for W_2_C, WS_2_, and W_2_C/WS_2_ electrodes by half-cell measurements. Table S1: Microstructural parameters of W_2_C. Table S2: Microstructural parameters of WS_2_. Table S3: Microstructural parameters of W_2_C/WS_2_. Table S4: Comparison of electrochemical parameters for different electrocatalysts. Table S5: HER catalytic performances TMDs and TMCs-based electrocatalysts. Table S6: Performances of TMDs- and TMCs-based electrodes for supercapacitors.
