# Supplementary material for: One-Pot Synthesis of W2C/WS2 Hybrid Nanostructures for Improved Hydrogen Evolution Reactions and Supercapacitors
Source: Nanomaterials (Basel). 2020 Aug 14;10(8):1597. doi: 10.3390/nano10081597 (PMC7466642; doi:10.3390/nano10081597)
Supplement: Supplementary file 1 [file nanomaterials-10-01597-s001.pdf]

nanomaterials

MDPI

Supporting Information

One-Pot Synthesis of W_2C/WS_2 Hybrid Nanostructures for Improved Hydrogen Evolution Reactions and Supercapacitors

Sajjad Hussain [1,2], Iqra Rabani [2], Dhanasekaran Vikraman [3], Asad Feroze [4], Muhammad Ali [5], Young-Soo Seo [2], Hyun-Seok Kim [3], Seung-Hyun Chun [4] and Jongwan Jung [1,2,*]

[1] Hybrid Materials Center (HMC), Sejong University, Seoul 05006, Korea; shussainawan@gmail.com
[2] Department of Nano and Advanced Materials Engineering, Sejong University, Seoul 05006, Korea; iqra.rabani@yahoo.com (I.R.); ysseo@sejong.ac.kr (Y.-S.S.)
[3] Division of Electronics and Electrical Engineering, Dongguk University-Seoul, Seoul 04620, Korea; v.j.dhanasekaran@gmail.com (D.V.); hyunseokk@dongguk.edu (H.-S.K.)
[4] Department of Physics, Sejong University, Seoul 05006, Korea; asadferoze55@gmail.com (A.F.); schun@sejong.ac.kr (S.-H.C.)
[5] Center of Research Excellence in Nanotechnology (CENT), King Fahd University of Petroleum and Minerals (KFUPM), Dhahran 31261, Saudi Arabia; muhammadaliaskari@gmail.com
* Correspondence: jwjung@sejong.ac.kr; Tel: +82-2-3408-3688; Fax: +82-2-3408-4342

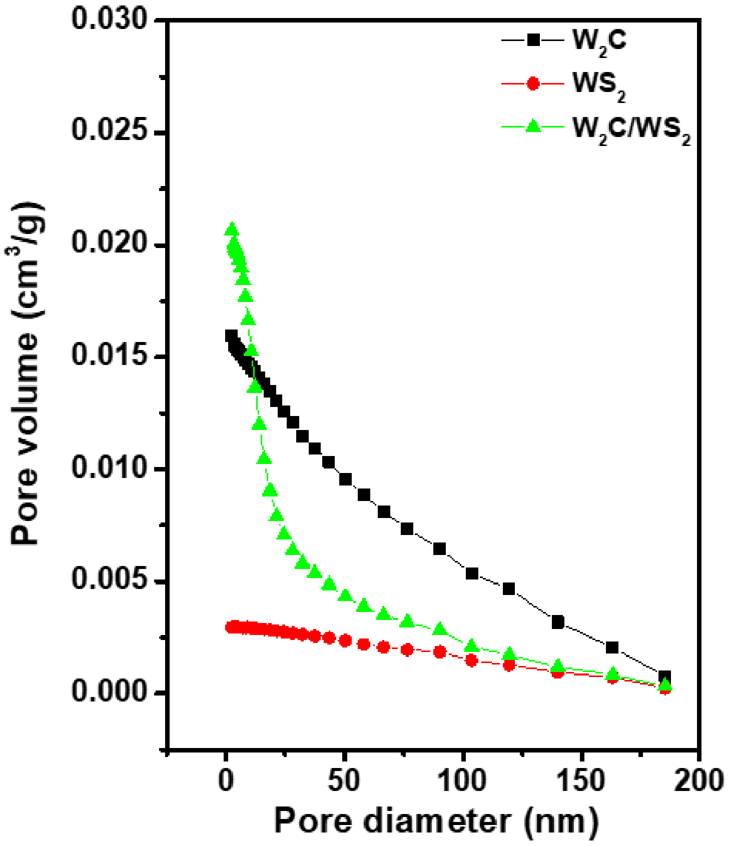

Figure S1. Pore diameter versus pore volume variations for the nanostructures.

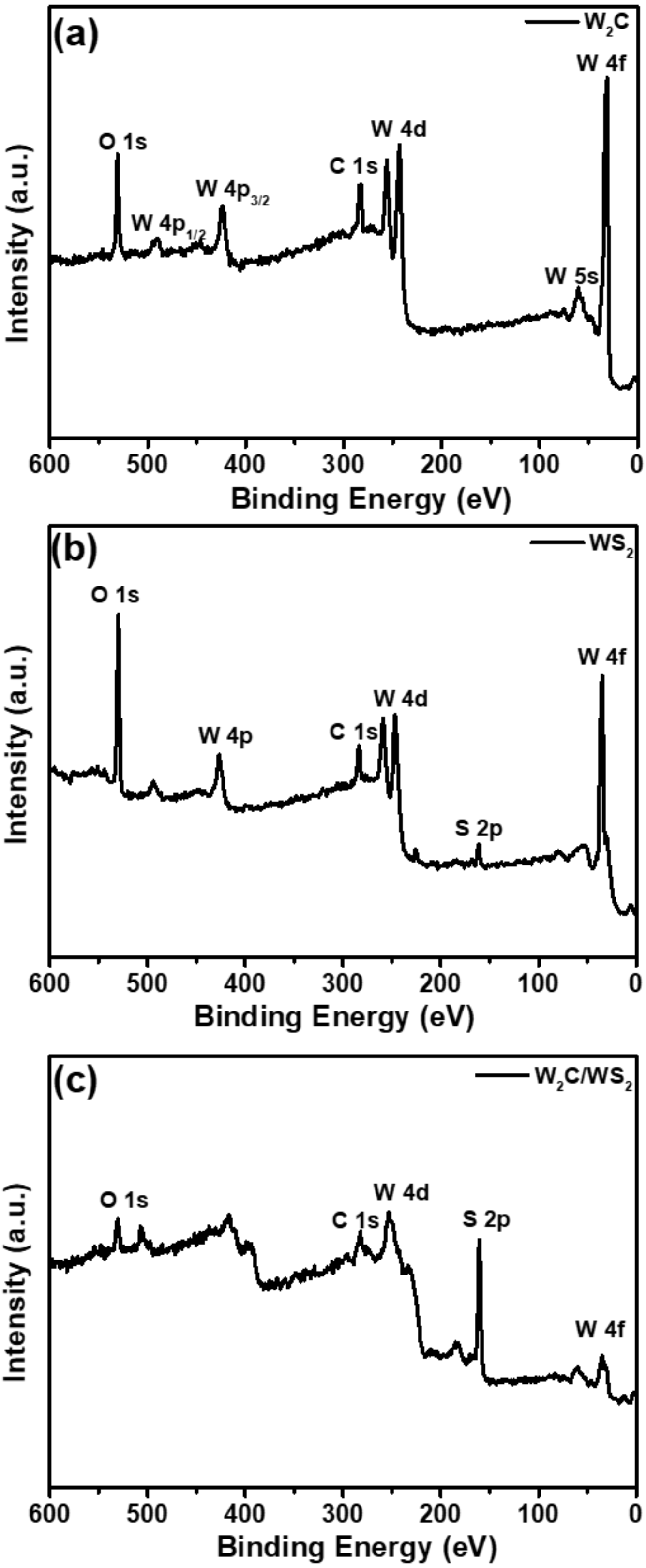

Figure S2. Survey XPS spectrum of **(a)** W_2C; **(b)** WS_2; **(c)** W_2C/WS_2.

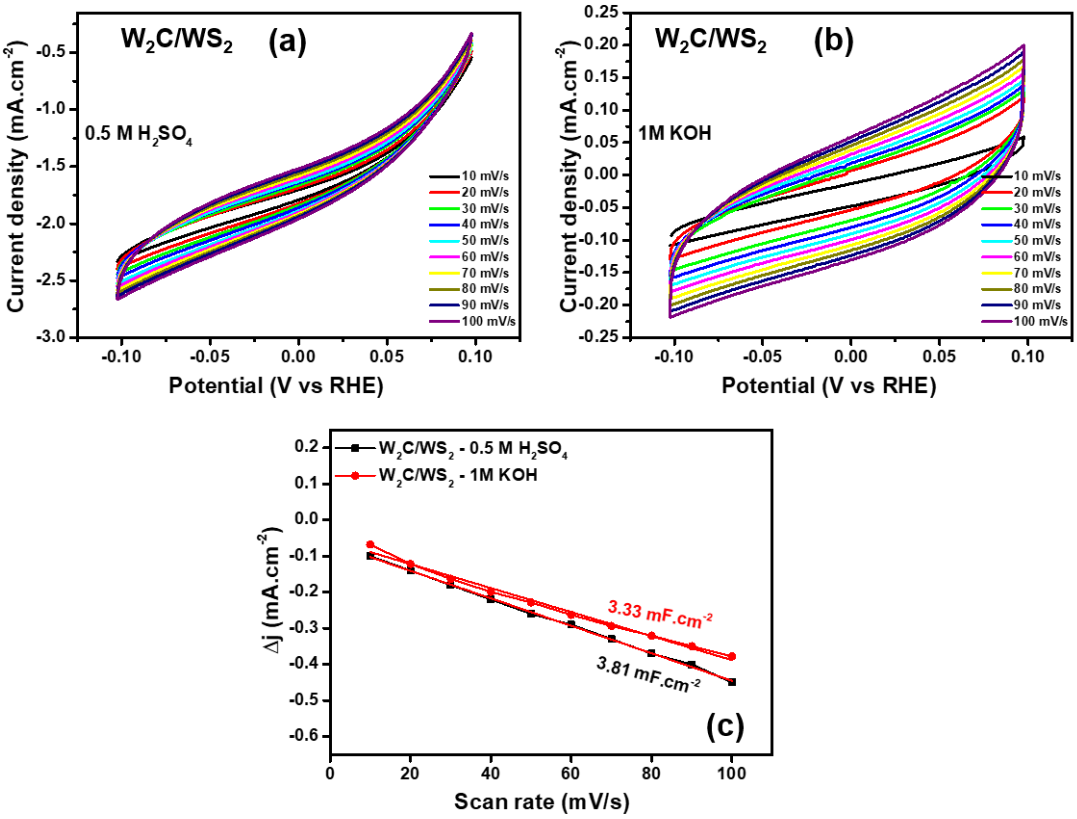

Figure S3. CV spectra in the non-Faradaic region with different scan rates for W_2C/WS_2 hybrid HER electrodes in **(a)** 0.5 M H_2SO_4 and **(b)** 1M KOH electrolyte and **(c)** their current differences.

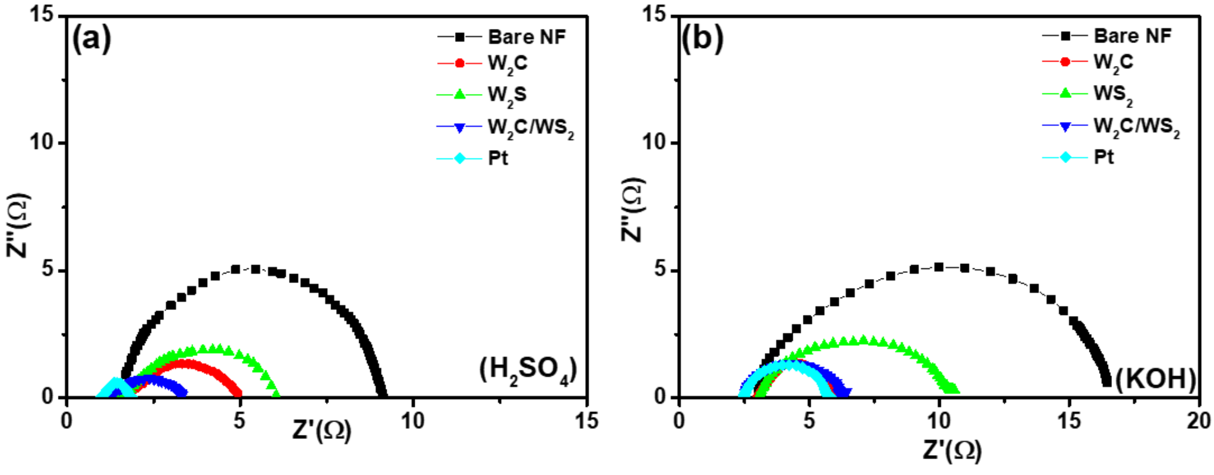

Figure S4. EIS spectra of Pt, W_2C, WS_2, and W_2C/WS_2 hybrids electrodes in **(a)** 0.5 M H_2SO_4 and **(b)** 1M KOH electrolyte.

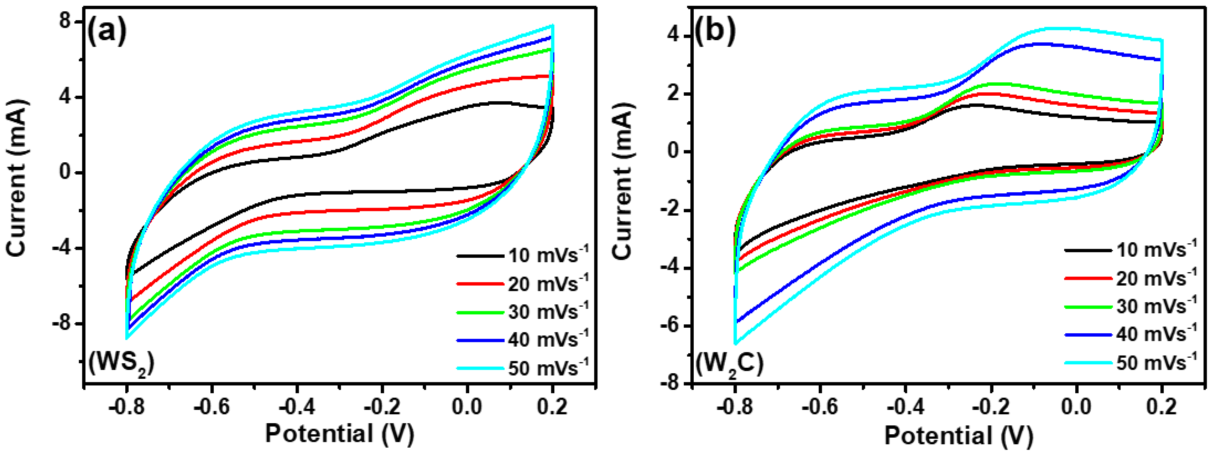

Figure S5. CV curves for the **(a)** WS_2 and **(b)** W_2C electrodes at various scan rates using half-cell (10-50 mV s^{-1}).

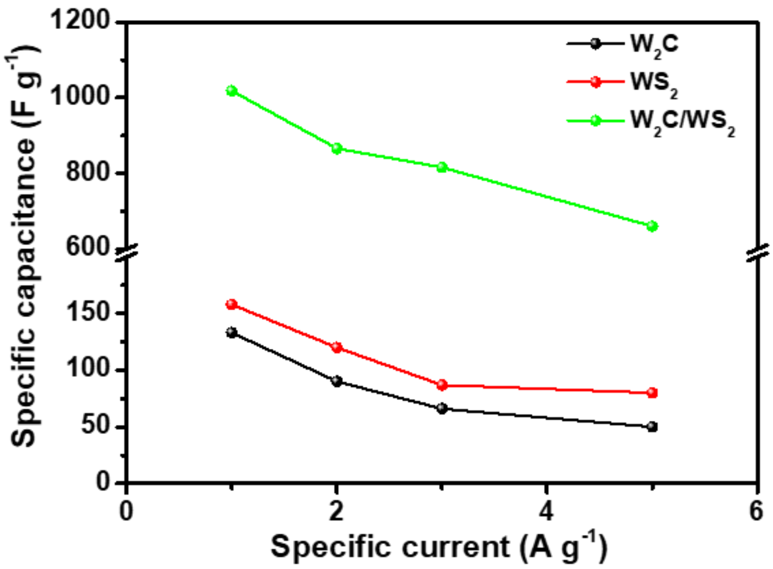

Figure S6. The specific capacitance variations at different current densities for W_2C, WS_2 and W_2C/WS_2 electrodes by half-cell measurements.

Table 1. Microstructural parameters of W_2C.

Lattice Plane	FWHM (°)	Crystallite Size (nm)	d-spacing (nm)
020	0.340	24.2	3.001
002	0.325	25.5	2.601
220	0.352	24.7	1.863
041	0.410	22.8	1.448
123	0.402	23.5	1.415
004	0.486	20.3	1.289
142	0.402	25.1	1.254
322	0.581	17.4	1.232

Table S2. Microstructural parameters of WS_2.

Lattice Plane	FWHM (°)	Crystallite Size (nm)	d-spacing (nm)
002	0.517	15.4	6.267
101	1.28	6.5	2.690
103	0.201	42.0	2.279
105	0.439	19.8	1.839
112	0.943	9.7	1.532
114	0.486	19.5	1.402
200	0.523	18.2	1.384

Table 3. Microstructural parameters of W_2C/WS_2.

Phase	Lattice Plane	FWHM (°)	Crystallite Size (nm)	d-spacing (nm)
WS_2	002	0.765	10.4	6.159
	101	1.120	7.4	2.714
	103	0.956	8.8	2.281
	110	0.907	10.0	1.570
W_2C	020	0.283	29.1	2.981
	002	0.341	24.4	2.591
	220	0.362	24.0	1.877
	041	0.450	20.8	1.450
	123	0.444	21.2	1.427
	004	0.362	27.2	1.291
	142	0.620	16.1	1.256
	322	0.575	17.6	1.234

Table S4. Comparison of electrochemical parameters for different electrocatalysts.

Electrolyte	Electrocatalysts	Overpotential (mV vs RHE) @ 10 mA·cm^{-2}	Tafel Slope (mV·dec^{-1})	Exchange Current Density (j_0, mA·cm^{-2})
**0.5 M H_2SO_4**	Pt/C	51	36	1.03
	W_2C/WS_2	133	70	1.02
	WS_2	242	138	0.549
	W_2C	171	86	0.194
	Bare NF	450	168	0.022
**1 M KOH**	Pt/C	48	48	1.08
	W_2C/WS_2	105	84	0.93
	WS_2	189	127	0.382
	W_2C	123	141	0.717
	Bare NF	292	148	0.048

Table S5. HER catalytic performances TMDs and TMCs-based electrocatalysts.

Electrocatalyst	Electrolyte	η (mV)	Tafel Slope (mV·dec^{-1})	j_0 (mA·cm^{-2})	Ref
W_2C/WS_2	0.5 M H_2SO_4	133 @ 10 mA/cm^2	70	1.02	This work
W_2C/WS_2	1.0 M KOH	105 @ 10 mA/cm^2	84	0.93	This work
WS_2/W_2C heterostructure	0.5 M H_2SO_4 & 1M KOH	126 & 205@ 10 mA/cm^2	68 & 72	0.501	[1]
W−W_2C/CNT	0.5 M H_2SO_4 & 1M KOH	155 & 147@ 10 mA/cm^2	63 & 64	-	[2]
p-WC_x NWs/CC	0.5 M H_2SO_4 & 1M KOH	118 & 122@ 10 mA/cm^2	56 & 56	0.165	[3]

W_2C@CNT-S hybrid	0.5 M H_2SO_4 & 1M KOH	174 & 148@ 10 mA/cm^2	57.3 & 56.2	-	[4]
Mo_2C/CNT	0.1 M $HClO_4$	250@ 10 mA/cm^2	251	1.43	[5]
Mo_2C/Ketjenblack carbon (KB)	1 M KOH	210@ 10 mA/cm^2	48	-	[6]
P-W_2C@NC	0.5 M H_2SO_4	89@ 10 mA/cm^2	53	0.316	[7]
W/BrN	0.5 M H_2SO_4 & 1M KOH	148 & 194@ 10 mA/cm^2	68 & 72	-	[8]
WC/W_2C heterojunction	0.5 M H_2SO_4	69@ 10 mA/cm^2	52	0.361	[9]
Mo_2C QD/NGCL	1 M KOH	111@ 10 mA/cm^2	57.8	-	[10]
W_xC@WS_2 Nanostructure	0.5 M H_2SO_4	146@ 10 mA/cm^2	61	0.042	[11]
Mo_2C/N-CNT	1 M KOH	257@ 10 mA/cm^2	77	10	[12]
W_2C@GL	0.5 M H_2SO_4	135@ 10 mA/cm^2	68	0.24	[13]
MoS_2/$Ti_3C_2T_x$ hybrid	0.5 M H_2SO_4	152@ 10 mA/cm^2	70	0.6165	[14]
CoS_2@WS_2	0.5 M H_2SO_4	97.2@ 10 mA/cm^2	66	-	[15]
Carbon coated cobalt– tungsten carbide Co_6W_6C	1 M KOH	73@ 10 mA/cm^2	25	-	[16]
MoS_2/ Mo_2C-NCNTs	0.5 M H_2SO_4	145@ 10 mA/cm^2	69	0.021	[17]
WC-CNTs	0.5 M H_2SO_4 & 1M KOH	145 &137@ 10 mA/cm^2	72 & 106	-	[18]
MoSSe@rGO composite	0.5 M H_2SO_4	135@5 mA/cm^2	51		[19]
Nano MoC@graphite shell	0.5 M H_2SO_4 &1 M KOH	124 & 77@ 10 mA/cm^2	43 & 50	-	[20]
α-Mo_2C nanoparticles	0.5 M H_2SO_4 &1 M KOH	198 & 176@ 10 mA/cm^2	56 & 58	-	[21]
MoS_2-Co nanoboxes	0.5 M H_2SO_4	155@ 10 mA/cm^2	55	0.013	[22]
MoC-Mo_2C HNWs	0.5 M H_2SO_4 &1 M KOH	126 & 120@ 10 mA/cm^2	43 & 42	-	[23]
$MoS_{2x}Se_{2(1-x)}$	0.5 M H_2SO_4	188@ 10 mA/cm^2	43	-	[24]
CNT-MoSeS	0.5 M H_2SO_4	174@ 10 mA/cm^2	40	-	[25]
Mo_2C@NC	0.5 M H_2SO_4 &1 M KOH	124 & 60@ 10 mA/cm^2	60	-	[26]
S-$MoSe_2$@GGNR hybrids	0.5 M H_2SO_4	153@ 10 mA/cm^2	46	-	[27]
1T/2HMoS_2	0.5 M H_2SO_4	220@ 10 mA/cm^2	61	0.443	[28]
N-doped Mo carbide and phosphide Hybrids (N@MoPCx)	0.5 M H_2SO_4	108@ 10 mA/cm^2	69.4	0.3424	[29]
$WS_{2(1-x)}$ Se_{2x} NTs	0.5 M H_2SO_4	298@ 10 mA/cm^2	105	0.029	[30]
FeS_2-RGO hybrid	0.5 M H_2SO_4	226@ 10 mA/cm^2	61	0.25~0.65	[31]
Co/N, B co doped ultrathin carbon cages (Co@BCN)	1 M KOH	183@ 10 mA/cm^2	73.2	0.103	[32]

W_2C/graphene nanoplatelets (GnP)	0.1m $HClO_4$	186@ 10 mA/cm^2	64.7	0.024	[33]
N-doped Mo_2C	0.5 M H_2SO_4 &1 M KOH	177.5 & 160.4@ 10 mA/cm^2	59.6 & 48.9	-	[34]

Table S6. Performances of TMDs and TMCs-based electrodes for supercapacitors.

**Electrode Materials**	**Electrolyte**	**Specific Capacitance**	**Capacitance Retention (%)/Cycle**	**Ref**
W_2C/WS_2 hybrid	1.0 M KOH	1018 F·g^{-1} @ 1.0 A·g^{-1}	94/5000	This work
WS_2/RGO hybrids	1.0 M Na_2SO_4	350 F/g @ 2mV/s	-	[35]
PANI-MoS_2 nanocomposite	1 M H_2SO_4	612 Fg^{-1} @ 0.2 A g^{-1}	93/2000	[36]
WS_2-MWCNTs/PANI	1 M H_2SO_4	760.1 Fg^{-1} @ 1 A g^{-1}	82.5/2000	[37]
VSe_2/carbon-nanotube		1854 uF / cm^2	90/10000	[38]
$TaSe_2$ Nanobelts	1 M KCl	835 mF cm^{-2} @ 2 mV s^{-1}	98.7/10000	[39]
MoS_2-NH_2/PANI nanosheets	1 M H_2SO_4	326.4 F g^{-1} at 0.5 A g^{-1}	96.5/10000	[40]
MoS_2 and WS_2 nanoparticles	3 M KOH	1531.2 & 1439.5 F g^{-1} at 5 mA cm^{-2}	81.6 & 74.4/2000	[41]
MXene/polypyrrole composite	1 M H_2SO_4	416 F g^{-1} at 0.5 A g^{-1}	86.4/5000	[42]
WS_2 QDs	H_3PO_4-PVA	28 mF cm^{-2} @ 0.1 mA cm^{-2}	80/10000	[43]
MoS_2/CNS	1 M Na_2SO_4	231 F g^{-1} at 1 A g^{-1}	-	[44]
MoS_2/RCF composite	1 M Na_2SO_4	225 F g^{-1} at 0.5 A g^{-1}	81/2000	[45]
1T′ -$MoTe_2$	2 M KOH	1393 F g^{-1} at A g^{-1}	98/1000	[46]
MoS_2/graphene nanobelts	1 M Na_2SO_4	445.71 F g^{-1} at 0.8 A g^{-1}	96.7/1000	[47]
MoS_2/rGO	1 M H_2SO_4	306 F g^{-1} at 0.5 A g^{-1}	-	[48]
WS_2/CFC	1 M KCl	399 F g^{-1} at 1 A g^{-1}	99/500	[49]
WS_2 nanosheets	PVA/LiCl gel	0.93 F cm^{-2} at 4 mA cm^{-2}	82/10000	[50]
$MoSe_2$	2 M KOH	243 F g^{-1} at 0.5 A g^{-1}	90.3/100	[51]
VS_2/MWCNTs	PVA-$LiClO_4$	182 F g^{-1} at 2 mV/s	93.2/5000	[52]
$MnO_2/Ti_3C_2T_x$ hybrid	1.0 M KOH	212.1 F g^{-1} @1 A g^{-1}	88/4000	[53]

References

1. Li, Y.; Wu, X.; Zhang, H.; Zhang, J. Interface designing over WS_2/W_2C for enhanced hydrogen evolution catalysis. *ACS Applied Energy Materials* **2018**, *1*, 3377-3384.
2. Hu, Y.; Yu, B.; Ramadoss, M.; Li, W.; Yang, D.; Wang, B.; Chen, Y. Scalable synthesis of heterogeneous W-W_2C nanoparticles embedded cnt networks for boosted hydrogen evolution reaction in both acidic and alkaline media. *ACS Sustainable Chemistry & Engineering* **2019,** *7*, 10016-10024.
3. Ren, B.; Li, D.; Jin, Q.; Cui, H.; Wang, C. Novel porous tungsten carbide hybrid nanowires on carbon cloth for high-performance hydrogen evolution. *Journal of Materials Chemistry A* **2017**, *5*, 13196-13203.
4. Hu, Y.; Yu, B.; Li, W.; Ramadoss, M.; Chen, Y. W_2C nanodot-decorated cnt networks as a highly efficient and stable electrocatalyst for hydrogen evolution in acidic and alkaline media. *Nanoscale* **2019**, *11*, 4876-4884.
5. Šljukić, B.; Vujković, M.; Amaral, L.; Santos, D.; Rocha, R.; Sequeira, C.; Figueiredo, J.L. Carbon-supported Mo_2C electrocatalysts for hydrogen evolution reaction. *Journal of Materials Chemistry A* **2015**, *3*, 15505-15512.

6. Wang, D.; Wang, J.; Luo, X.; Wu, Z.; Ye, L. In situ preparation of Mo_2C nanoparticles embedded in ketjenblack carbon as highly efficient electrocatalysts for hydrogen evolution. *ACS Sustainable Chemistry & Engineering* **2017**, *6*, 983-990.
7. Yan, G.; Wu, C.; Tan, H.; Feng, X.; Yan, L.; Zang, H.; Li, Y. N-carbon coated PW_2C composite as efficient electrocatalyst for hydrogen evolution reactions over the whole ph range. *Journal of Materials Chemistry A* **2017**, *5*, 765-772.
8. Li, Q.; Han, C.; Ma, X.; Wang, D.; Xing, Z.; Yang, X. Bromine and nitrogen co-doped tungsten nanoarrays to enable hydrogen evolution at all ph values. *Journal of Materials Chemistry A* **2017**, *5*, 17856-17861.
9. Zhang, L.-N.; Ma, Y.-Y.; Lang, Z.-L.; Wang, Y.-H.; Khan, S.U.; Yan, G.; Tan, H.-Q.; Zang, H.-Y.; Li, Y.-g. Ultrafine cable-like WC/W_2C heterojunction nanowires covered by graphitic carbon towards highly efficient electrocatalytic hydrogen evolution. *Journal of Materials Chemistry A* **2018**, *6*, 15395-15403.
10. Pu, Z.; Wang, M.; Kou, Z.; Amiinu, I.S.; Mu, S. Mo_2C quantum dot embedded chitosan-derived nitrogen-doped carbon for efficient hydrogen evolution in a broad ph range. *Chemical Communications* **2016**, *52*, 12753-12756.
11. Wang, F.; He, P.; Li, Y.; Shifa, T.A.; Deng, Y.; Liu, K.; Wang, Q.; Wang, F.; Wen, Y.; Wang, Z. Interface engineered $W_xC@WS_2$ nanostructure for enhanced hydrogen evolution catalysis. *Advanced Functional Materials* **2017**, *27*, 1605802.
12. Zhang, K.; Zhao, Y.; Fu, D.; Chen, Y. Molybdenum carbide nanocrystal embedded n-doped carbon nanotubes as electrocatalysts for hydrogen generation. *Journal of Materials Chemistry A* **2015**, *3*, 5783-5788.
13. Zhou, Y.; Ma, R.; Li, P.; Chen, Y.; Liu, Q.; Cao, G.; Wang, J. Ditungsten carbide nanoparticles encapsulated by ultrathin graphitic layers with excellent hydrogen-evolution electrocatalytic properties. *Journal of Materials Chemistry A* **2016**, *4*, 8204-8210.
14. Liu, J.; Liu, Y.; Xu, D.; Zhu, Y.; Peng, W.; Li, Y.; Zhang, F.; Fan, X. Hierarchical "nanoroll" like $MoS_2/Ti_3C_2T_x$ hybrid with high electrocatalytic hydrogen evolution activity. *Applied Catalysis B: Environmental* **2019**, *241*, 89-94.
15. Zhou, X.; Yang, X.; Li, H.; Hedhili, M.N.; Huang, K.-W.; Li, L.-J.; Zhang, W. Symmetric synergy of hybrid CoS_2–WS_2 electrocatalysts for the hydrogen evolution reaction. *Journal of Materials Chemistry A* **2017**, *5*, 15552-15558.
16. Liu, Y.; Li, G.-D.; Yuan, L.; Ge, L.; Ding, H.; Wang, D.; Zou, X. Carbon-protected bimetallic carbide nanoparticles for a highly efficient alkaline hydrogen evolution reaction. *Nanoscale* **2015**, *7*, 3130-3136.
17. Zhang, K.; Zhao, Y.; Zhang, S.; Yu, H.; Chen, Y.; Gao, P.; Zhu, C. MoS_2 nanosheet/Mo_2C-embedded n-doped carbon nanotubes: Synthesis and electrocatalytic hydrogen evolution performance. *Journal of Materials Chemistry A* **2014**, *2*, 18715-18719.
18. Fan, X.; Zhou, H.; Guo, X. WC nanocrystals grown on vertically aligned carbon nanotubes: An efficient and stable electrocatalyst for hydrogen evolution reaction. *ACS Nano* **2015**, *9*, 5125-5134.
19. Konkena, B.; Masa, J.; Xia, W.; Muhler, M.; Schuhmann, W. MoSSe@reduced graphene oxide nanocomposite heterostructures as efficient and stable electrocatalysts for the hydrogen evolution reaction. *Nano Energy* **2016**, *29*, 46-53.
20. Shi, Z.; Wang, Y.; Lin, H.; Zhang, H.; Shen, M.; Xie, S.; Zhang, Y.; Gao, Q.; Tang, Y. Porous nanoMoC@ graphite shell derived from a MOFs-directed strategy: An efficient electrocatalyst for the hydrogen evolution reaction. *Journal of Materials Chemistry A* **2016**, *4*, 6006-6013.
21. Ma, L.; Ting, L.R.L.; Molinari, V.; Giordano, C.; Yeo, B.S. Efficient hydrogen evolution reaction catalyzed by molybdenum carbide and molybdenum nitride nanocatalysts synthesized via the urea glass route. *Journal of Materials Chemistry A* **2015**, *3*, 8361-8368.
22. Chen, G.; Dong, W.F.; Li, B.L.; Deng, Y.H.; Wang, X.H.; Zhang, X.F.; Luo, H.Q.; Li, N.B. Cobalt incorporated MoS_2 hollow structure with rich out-of-plane edges for efficient hydrogen production. *Electrochimica Acta* **2018**, *276*, 81-91.
23. Lin, H.; Shi, Z.; He, S.; Yu, X.; Wang, S.; Gao, Q.; Tang, Y. Heteronanowires of MoC–Mo_2C as efficient electrocatalysts for hydrogen evolution reaction. *Chemical science* **2016**, *7*, 3399-3405.
24. Zhen, C.; Zhang, B.; Zhou, Y.; Du, Y.; Xu, P. Hydrothermal synthesis of ternary $MoS_{2x}Se_{2(1-x)}$ nanosheets for electrocatalytic hydrogen evolution. *Inorganic Chemistry Frontiers* **2018**, *5*, 1386-1390.
25. Yang, J.; Liu, Y.; Shi, C.; Zhu, J.; Yang, X.; Liu, S.; Li, L.; Xu, Z.; Zhang, C.; Liu, T. Carbon nanotube with vertical 2D molybdenum sulphoselenide nanosheet arrays for boosting electrocatalytic hydrogen evolution. *ACS Applied Energy Materials* **2018**, *1*, 7035-7045.

26. Liu, Y.; Yu, G.; Li, G.D.; Sun, Y.; Asefa, T.; Chen, W.; Zou, X. Coupling Mo_2C with nitrogen-rich nanocarbon leads to efficient hydrogen-evolution electrocatalytic sites. *Angewandte Chemie International Edition* **2015**, *54*, 10752-10757.
27. Fan, W.; Wang, D.; Sun, Z.; Ling, X.Y.; Liu, T. Graphene/graphene nanoribbon aerogels decorated with s-doped $MoSe_2$ nanosheets as an efficient electrocatalyst for hydrogen evolution. *Inorganic Chemistry Frontiers* **2019**, *6*, 1209-1216.
28. Liu, Z.; Gao, Z.; Liu, Y.; Xia, M.; Wang, R.; Li, N. Heterogeneous nanostructure based on 1T-phase MoS_2 for enhanced electrocatalytic hydrogen evolution. *ACS applied materials & interfaces* **2017**, *9*, 25291-25297.
29. Huang, Y.; Ge, J.; Hu, J.; Zhang, J.; Hao, J.; Wei, Y. Nitrogen-doped porous molybdenum carbide and phosphide hybrids on a carbon matrix as highly effective electrocatalysts for the hydrogen evolution reaction. *Advanced Energy Materials* **2018**, *8*, 1701601.
30. Xu, K.; Wang, F.; Wang, Z.; Zhan, X.; Wang, Q.; Cheng, Z.; Safdar, M.; He, J. Component-controllable $WS_{2(1-x)}Se_{2x}$ nanotubes for efficient hydrogen evolution reaction. *Acs Nano* **2014**, *8*, 8468-8476.
31. Jiang, J.; Zhu, L.; Chen, H.; Sun, Y.; Qian, W.; Lin, H.; Han, S. Highly active and stable electrocatalysts of FeS_2–reduced graphene oxide for hydrogen evolution. *Journal of materials science* **2019**, *54*, 1422-1433.
32. Zhang, H.; Ma, Z.; Duan, J.; Liu, H.; Liu, G.; Wang, T.; Chang, K.; Li, M.; Shi, L.; Meng, X. Active sites implanted carbon cages in core–shell architecture: Highly active and durable electrocatalyst for hydrogen evolution reaction. *ACS Nano* **2015**, *10*, 684-694.
33. Chen, W.F.; Schneider, J.M.; Sasaki, K.; Wang, C.H.; Schneider, J.; Iyer, S.; Iyer, S.; Zhu, Y.; Muckerman, J.T.; Fujita, E. Tungsten carbide–nitride on graphene nanoplatelets as a durable hydrogen evolution electrocatalyst. *ChemSusChem* **2014**, *7*, 2414-2418.
34. Jiang, R.; Fan, J.; Hu, L.; Dou, Y.; Mao, X.; Wang, D. Electrochemically synthesized n-doped molybdenum carbide nanoparticles for efficient catalysis of hydrogen evolution reaction. *Electrochimica Acta* **2018**, *261*, 578-587.
35. Ratha, S.; Rout, C.S. Supercapacitor electrodes based on layered tungsten disulfide-reduced graphene oxide hybrids synthesized by a facile hydrothermal method. *ACS applied materials & interfaces* **2013**, *5*, 11427-11433.
36. Raghu, M.; Kumar, K.Y.; Rao, S.; Aravinda, T.; Prasanna, B.; Prashanth, M. Fabrication of polyaniline–few-layer MoS_2 nanocomposite for high energy density supercapacitors. *Polymer Bulletin* **2018**, *75*, 4359-4375.
37. Gao, J.; Ma, Y.; Li, J.; Fan, J.; Shi, P.; Xu, Q.; Min, Y. Free-standing WS_2-MWCNTs hybrid paper integrated with polyaniline for high-performance flexible supercapacitor. *Journal of Nanoparticle Research* **2018**, *20*, 298.
38. Wang, C.; Wu, X.; Xu, H.; Zhu, Y.; Liang, F.; Luo, C.; Xia, Y.; Xie, X.; Zhang, J.; Duan, C. VSe_2/carbon-nanotube compound for all solid-state flexible in-plane supercapacitor. *Applied Physics Letters* **2019**, *114*, 023902.
39. Wang, M.; Zhang, L.; Zhong, Y.; Huang, M.; Zhen, Z.; Zhu, H. In situ electrodeposition of polypyrrole onto $TaSe_2$ nanobelts quasi-arrays for high-capacitance supercapacitor. *Nanoscale* **2018**, *10*, 17341-17346.
40. Zeng, R.; Li, Z.; Li, L.; Li, Y.; Huang, J.; Xiao, Y.; Yuan, K.; Chen, Y. Covalent connection of polyaniline with MoS_2 nanosheets towards ultrahigh rate capability supercapacitors. *ACS Sustainable Chemistry & Engineering* **2019,** *7*, 11540-11549.
41. Nagaraju, C.; Gopi, C.V.M.; Ahn, J.-W.; Kim, H.-J. Hydrothermal synthesis of MoS_2 and WS_2 nanoparticles for high-performance supercapacitor applications. *New Journal of Chemistry* **2018**, *42*, 12357-12360.
42. Jian, X.; He, M.; Chen, L.; Zhang, M.-m.; Li, R.; Gao, L.-j.; Fu, F.; Liang, Z.-h. Three-dimensional carambola-like MXene/polypyrrole composite produced by one-step co-electrodeposition method for electrochemical energy storage. *Electrochimica Acta* **2019,** *318*, 820-827.
43. Ghorai, A.; Midya, A.; Ray, S.K. Superior charge storage performance of WS_2 quantum dots in a flexible solid state supercapacitor. *New Journal of Chemistry* **2018**, *42*, 3609-3613.
44. Khawula, T.N.; Raju, K.; Franklyn, P.J.; Sigalas, I.; Ozoemena, K.I. Symmetric pseudocapacitors based on molybdenum disulfide (MoS_2)-modified carbon nanospheres: Correlating physicochemistry and synergistic interaction on energy storage. *Journal of Materials Chemistry A* **2016**, *4*, 6411-6425.
45. Zhao, C.; Zhou, Y.; Ge, Z.; Zhao, C.; Qian, X. Facile construction of MoS_2/RCF electrode for high-performance supercapacitor. *Carbon* **2018**, *127*, 699-706.
46. Liu, M.; Wang, Z.; Liu, J.; Wei, G.; Du, J.; Li, Y.; An, C.; Zhang, J. Synthesis of few-layer 1T′-$MoTe_2$ ultrathin nanosheets for high-performance pseudocapacitors. *Journal of Materials Chemistry A* **2017**, *5*, 1035-1042.
47. Jia, Y.; Wan, H.; Chen, L.; Zhou, H.; Chen, J. Hierarchical nanosheet-based MoS_2/graphene nanobelts with high electrochemical energy storage performance. *Journal of Power Sources* **2017**, *354*, 1-9.

48. Ji, H.; Hu, S.; Jiang, Z.; Shi, S.; Hou, W.; Yang, G. Directly scalable preparation of sandwiched MoS_2/graphene nanocomposites via ball-milling with excellent electrochemical energy storage performance. *Electrochimica Acta* **2019**, *299*, 143-151.
49. Shang, X.; Chi, J.-Q.; Lu, S.-S.; Gou, J.-X.; Dong, B.; Li, X.; Liu, Y.-R.; Yan, K.-L.; Chai, Y.-M.; Liu, C.-G. Carbon fiber cloth supported interwoven WS_2 nanosplates with highly enhanced performances for supercapacitors. *Applied Surface Science* **2017**, *392*, 708-714.
50. Liu, S.; Zeng, Y.; Zhang, M.; Xie, S.; Tong, Y.; Cheng, F.; Lu, X. Binder-free WS_2 nanosheets with enhanced crystallinity as a stable negative electrode for flexible asymmetric supercapacitors. *Journal of Materials Chemistry A* **2017**, *5*, 21460-21466.
51. Gao, Y.-P.; Huang, K.-J.; Shuai, H.-L.; Liu, L. Synthesis of sphere-feature molybdenum selenide with enhanced electrochemical performance for supercapacitor. *Materials Letters* **2017**, *209*, 319-322.
52. Pandit, B.; Karade, S.S.; Sankapal, B.R. Hexagonal VS_2 anchored MWCNTs: First approach to design flexible solid-state symmetric supercapacitor device. *ACS applied materials & interfaces* **2017**, *9*, 44880-44891.
53. Rakhi, R.B.; Ahmed, B.; Anjum, D.; Alshareef, H.N. Direct chemical synthesis of MnO_2 nanowhiskers on transition-metal carbide surfaces for supercapacitor applications. *ACS applied materials & interfaces* **2016**, *8*, 18806-18814.
